# Results from a pharmacy-based patient survey on the use of a fixed combination analgesic containing acetylsalicylic acid, paracetamol and caffeine by self-diagnosing and self-treating patients

**DOI:** 10.1186/s40064-016-2369-0

**Published:** 2016-06-14

**Authors:** Charly Gaul, Heidemarie Gräter, Thomas Weiser

**Affiliations:** Migraine and Headache Clinic Königstein, Ölmühlweg 31, 61462 Königstein im Taunus, Germany; Boehringer Ingelheim Pharma GmbH & Co. KG, Ingelheim, Germany

**Keywords:** Acetylsalicylic acid, Paracetamol, Caffeine, Headache, Migraine

## Abstract

**Background:**

Patients suffering from migraine or tension-type headache (TTH) often treat their complaints with over-the-counter (OTC) medications. Fixed dose combinations of acetylsalicylic acid, paracetamol and caffeine (APC) are among the most commonly used analgesics, and their efficacy for treating acute headache pain has been well demonstrated. This investigation was run to better characterize patients who treat their headache with OTC APC combinations, as well as treatment effects.

**Methods:**

A pharmacy-based patient survey in 164 German pharmacies was performed. Patients (age ≥18 years) who purchased APC analgesics (of the brand Thomapyrin^®^) were handed a questionnaire, which had to be filled out at patients own discretion after taking the medication. Demographics, pain characteristics and perceived efficacy and tolerability data were analysed with descriptive statistics.

**Results:**

Questionnaires from 1298 patients were analysed, of whom 71.9 % were females and 28.1 % were males. Headache patients were assigned to TTH or migraine according to diagnosis criteria of the International Headache Classification-II (ICHD-II), with 828 patients for TTH and 206 for migraine. About one third of patients in the subgroup migraine did not report their pain as migraine. Nausea, photophobia/phonophobia turned out to be the most distinguishing feature between migraine and TTH. The main reasons for purchasing the product were recommendation by the pharmacists (40.5 %) and/or friends or relatives (24.4 %). 74 % of TTH and 55 % of migraine patients reported onset of pain relief within the first 30 min. More than 90 % rated efficacy as well as tolerability to be “good” or “very good”.

**Conclusions:**

The main reason for purchasing APC products in the pharmacy are TTH or migraine. About a third of patients fulfilling the IHCD-II criteria for migraine failed to recognize their headache as migraine. This could be explained e.g. by patients’ misconceptions about their pain. Patients’ assessments of efficacy and tolerability showed that the investigated APC combinations are valuable for the treatment of TTH and migraine headache. These data complement those of randomized clinical studies on such preparations.

## Background

Fixed dose combinations of acetylsalicylic acid, paracetamol and caffeine are in widespread use for the treatment of episodic headaches. Typical over-the-counter (OTC) products contain 250 mg acetylsalicylic acid, 200 or 250 mg paracetamol and 50 mg caffeine per tablet (APC combination), with the recommended dose of one to two tablets every 4–8 h up to six tablets daily.

APC combinations are among the best investigated analgesics for the treatment of acute headaches in self-medication. In all controlled, randomized, placebo-controlled studies performed so far, this triple combination showed statistically significant and clinically relevant superiority over comparators, among them the mono-compounds acetylsalicylic acid, paracetamol, and ibuprofen, when used with the maximum recommended OTC dose, as well as the prescription-only migraine medication sumatriptan (Laska et al. 1983, 1984; Migliardi et al. 1994; Lipton et al. 1998; Diener et al. 2005; Goldstein et al. 2005, 2006). A meta-analysis of RCTs demonstrated the benefit of the APC combination for patients with episodic TTH even in those with pain rated severe. The combination was superior to placebo, as well as to paracetamol for the endpoints pain-free at 2 h, headache response at 2 h, and ability to return to daily activities (Diener et al. 2014). According to the evidence-based therapeutic guideline, the APC combination is a first-line treatment for the self-medication of episodic migraine and TTH (with “highlighted recommendation on the basis of the analysed comparative studies”), recommended by the German Neurological Society, as well as the headache societies in Germany, Austria and Switzerland (Haag et al. 2011).

This pharmacy-based patient survey collected data regarding the usage of two APC preparations in self-diagnosing and self-treating patients under daily life conditions. The aim of the study was to extend the current knowledge about characteristics of patients, their pain and pain treatment, as well as efficacy and tolerability of these fixed combination analgesics.

## Methods

This prospective, non-interventional survey was run in 164 German pharmacies between October 2013 and March 2014. Patients who purchased Thomapyrin^®^ CLASSIC or Thomapyrin^®^ INTENSIV [containing 250 mg acetylsalicylic acid, 200 (“CLASSIC”) or 250 mg paracetamol (“INTENSIV”) and 50 mg caffeine per tablet] and consented to participate were handed a questionnaire, which had to be filled out at patients own discretion after taking the medication, preferably on the same day. Patients were asked to send the completed questionnaire to the institute that analysed the data (Winicker Norimed GmbH, Germany) in a sealed envelope. Personal data enabling to identify a patient were not collected. Thus no formal ethical approval was necessary.

Preconditions for participation comprised age ≥18 years, purchase of one of the both APC medications for treatment of headache or any other pain, willingness and ability to independent, plausible and timely completion of the questionnaire. No exclusion criteria were defined.

The paper based questionnaire obtained—among others—information on sex, age, height, weight, frequency of pain within last 30 days, limitation of daily activity by pain (within the last 30 days), and reasons for purchasing this products.

Parameters related to last pain episode provoking the intake of the drug: first usage (yes/no), type of pain (headache, migraine, other), intensity of pain (numeric rating scale; scale of 0–10), time of pain event, time of first usage of the drug after onset of pain event; if applicable: time of repeated usage, time to onset of pain relief, subject’s assessment of efficacy, subject’s assessment of tolerability, willingness to recommend usage of Thomapyrin^®^ to others.

The characterization of a subject’s type of headache pain was based on the diagnostic criteria of the International Classification of Headache Disorders (ICHD-II, 2004), i.e. pain location [one-sided (1a), both-sided or diffuse (1b)]; pain characteristics [throbbing (2a), pressing/tightening (2b)]; whether pain increased due to physical strain [yes (3a), no (3b)]; occurrence of the accompanying symptoms nausea, photophobia or phonophobia [yes (4a), no (4b)]. A subject was assigned to subgroup “migraine” if (1a AND 2a AND 4a) or (1a AND 3a AND 4a) or (2a AND 3a AND 4a) were met. A subject was assigned to subgroup “TTH” if [1b AND 2b AND (3b OR 4b)] or (2b AND 3b AND 4b) or (1b AND 3b AND 4b) were met. Moreover, patients were asked to assign their headache to “migraine” or “headache” (i.e. all other non-migraine headaches). A subject was assigned to the subgroup “other”, if the subject’s diagnosis of “type of pain” was “other”. In case a subject could not be assigned unambiguously to any subgroup, the subject was omitted from the analyses pertaining to this subgroup.

### Safety

Any adverse drug reaction arising out of completed questionnaires had to be reported by the institute that collected and analysed the data to Boehringer Ingelheim and was processed accordingly.

### Data analysis

Data management and statistical analysis were performed using SAS, Version 9.2. As appropriate for the design of the study, only descriptive analyses and statistical modelling were performed. Numerical data were compared exploratively between subgroups using a Wilcoxon or Kruskal–Wallis test. Analyses were done for the total study population and for study subgroups separately.

## Results

### Study population

A total of 1302 patients completed the questionnaire. The data from four patients were excluded from the analysis, because three of them had no documented intake of the product, and one did not report the type of pain treated. Thus, the data of 1298 patients were available for the analysis. A summary of the main demographic variables is provided in Table 1.

**Table 1:** Demographics

Sex	
Male [n (%)]	365 (28.1)
Female [n (%)]	932 (71.9)
Age (years)	
Mean ± SD	39.2 ± 14.1
Median (range)	36 (16–88)
Height (cm)	
Mean ± SD	171.1 ± 9.2
Median (range)	170 (147–203)
Weight (kg)	
Mean ± SD	71.7 ± 14.9
Median (range)	69 (42–140)
BMI (kg/m^2^)	
Mean ± SD	24.4 ± 4.0
Median (range)	23.7 (14.9–47.3)

### Pain anamnesis

A total of 1284 patients reported a mean of 5.4 ± 5.5 days with any kind of pain during the last 30 days. Female patients reported overall more days with pain than males [5.6 ± 5.7 (n = 921) vs. 4.8 ± 5.0 (n = 362); p = 0.002]. Migraine patients reported more days with pain than TTH patients [6.7 ± 6.0 (n = 204) vs. 4.8 ± 5.0 (n = 818); p < 0.001].

Information on the number of days (during the last 30 day interval) where activities of daily living could not be performed due to pain was obtained from 1057 patients. In total, a mean of 1.5 ± 3.2 days was reported. Females reported slightly more days than male patients [1.6 ± 3.3 (n = 751) vs. 1.3 ± 2.9 (n = 305); p = 0.025], and migraine patients more than TTH patients [2.5 ± 3.6 (n = 206) vs. 1.1 ± 2.8 (n = 828); p = <0.001].

Mean baseline pain intensity before the intake of the medication was 5.9 ± 1.7. Migraine patients reported more severe pain (6.8 ± 1.6; n = 204) than TTH patients (5.6 ± 1.7; n = 824; p < 0.001); no difference in baseline pain between male and female patients was found.

Based on requested information about the last episode of headache, 828 patients fulfilled the IHCD-II criteria for TTH. 760 patients (91.8 %) of them documented non-migraine headache themselves, while 12 patients (1.4 %) documented migraine. The remaining 56 patients (6.8 %) did not clearly allocate their headache pain to one specific type.

A total of 206 patients were attributed to migraine. 111 patients (53.9 %) of them diagnosed migraine themselves, while 61 patients (29.6 %) documented non-migraine headache. The remaining 34 patients (16.5 %) did not clearly allocate their headache pain to one specific type. Thus, about one third of the patients fulfilling the IHCD-II criteria for migraine did not recognize their pain as migraine.

Thirty patients among all reported to suffer from migraine and non-migraine headache. The acute episode of nine of them (30.0 %) was assigned to TTH, 21 of them (70.0 %) to migraine.

The pain characteristics are shown in Table 2. These data show that e.g. 26.5 % of patients with migraine felt the pain to be “pressing/tightening”, and the same percentage of patients with TTH reported it to be “throbbing”. 91.2 % of patients with migraine versus 53.0 % with TTH reported increasing pain during physical strain. Nausea, photophobia/phonophobia were present in 95.6 % of patients with migraine (versus 10.4 % in TTH), and therefore turned out to be the most distinguishing feature between migraine and TTH.

**Table 2:** Pain characteristics/symptoms

	Headache (N = 828) n (%)	Migraine (N = 206) n (%)	p value
Location of pain			
One-sided	195 (23.8 %)	141 (68.4 %)	<0.001
Bilateral	351 (42.9 %)	39 (18.9 %)	
Diffuse, assignment of side not possible	273 (33.3 %)	26 (12.6 %)	
Missing values	9	–	
Pain characteristics			
Throbbing	217 (26.5 %)	145 (71.1 %)	<0.001
Pressing/tightening	595 (72.6 %)	54 (26.5 %)	
Both	7 (0.9 %)	5 (2.5 %)	
Missing values	9	2	
Increasing pain during physical strain			
No	383 (47.0 %)	18 (8.8 %)	<0.001
Yes	432 (53.0 %)	187 (91.2 %)	
Missing values	13	1	
Nausea/photophobia/phonophobia present?			
No	733 (89.6 %)	9 (4.4 %)	<0.001
Yes	85 (10.4 %)	196 (95.6 %)	
Missing values	10	1	

### Surveyed treatment

The reasons for buying the medication are shown in Table 3. In the majority of cases recommendations (by pharmacists, relatives or friends) triggered the purchase decision.

**Table 3:** Reasons for purchasing the medication

Recommended by pharmacist	526 (40.5 %)
Recommended by relative/friend	317 (24.4 %)
Known from advertising	295 (22.7 %)
Other	276 (21.3 %)
Recommended by physician	36 (2.8 %)

The median time between occurrence of the pain episode and intake of the medication was 45 min (n = 942), with no difference between male and female patients. However, migraine patients took the medication earlier than those suffering from TTH [median 30 min for migraine (n = 153) vs. 45 min for headache (n = 594) vs. 105 min for other pain (n = 163); p < 0.001]. Patients who took the APC combination for the first time waited longer than those who already knew the product [median of 90 min (n = 287) for first-time users vs. 35 min (n = 646) for experienced users; p < 0.001].

The mean dose at first intake was 1.4 ± 0.5 tablets, with a mean total of 1.8 ± 1.2 tablets for the entire pain event. The mean dose was slightly higher for migraine versus TTH patients (1.5 ± 0.6; n = 179 vs. 1.3 ± 0.5; n = 706; p < 0.001). Higher percentages of migraine patients took more than one dose during the pain episode (migraine: 59.8 % one, 24.6 % two, and 15.6 % three doses compared to 82.0, 12.7 and 5.4 % for TTH, p < 0.001). Consequently, the mean total number of tablets per pain episode was also higher for migraine patients [2.3 ± 1.4 (n = 206) for migraine vs. 1.6 ± 1.0 (n = 710) for TTH; p < 0.001].

### Parameters of efficacy

**Fig. 1:**
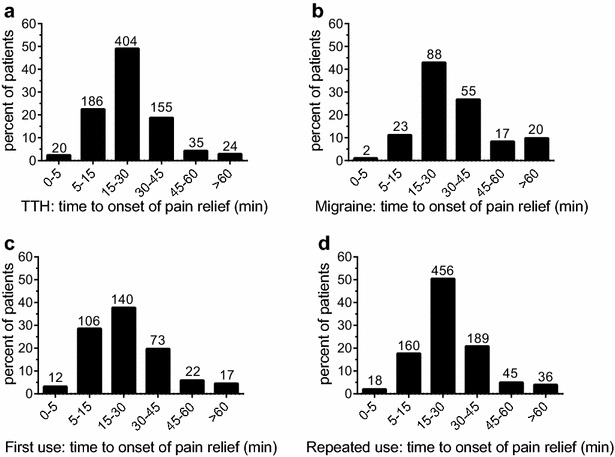
Time to onset of pain relief after intake of the APC combination. **a**, **b** Show the data for TTH and migraine patients. In both patient groups the majority reported onset of pain relief within 30 min. A higher proportion of patients who used the APC combination for the first time (**c**) reported onset of pain relief within the first 15 min after intake, compared to experienced APC users (**d**). See text for details. Numbers of patients for the respective groups are given above the* columns*

Fast onset of action of an analgesic is desired by patients and contributes to the overall impression of efficacy (Pageler et al. 2009). Onset of pain relief as reported by the patients was different for TTH and migraine (p < 0.001; Fig. 1a, b): e.g. 74 % of the TTH patients reported onset of pain relief within 30 min after intake of the medication, compared to 55 % of the migraine patients. Also previous experience with the medication, compared to first time usage, affected the perceived time to onset of action (p < 0.001; Fig. 1c, d). The same percentage of patients report onset of pain relief within the first 30 min after intake (70 %), however, a higher percentage of first-time users perceived pain relief within the first 15 min after intake (32 vs. 20 %).

**Fig. 2:**
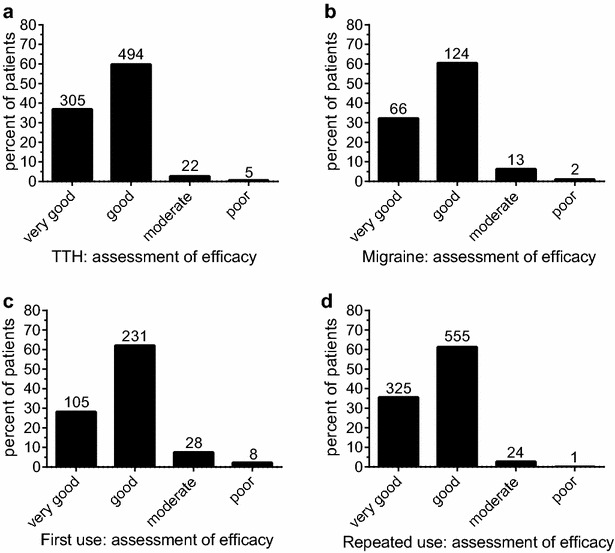
Patients’ assessment of efficacy. Assessment of efficacy for patients using the APC combination for the treatment of TTH (**a**) or migraine (**b**), as well as for patients taking the medication for the first time (**c**), compared to experienced users (**d**). See text for details. Numbers of patients for the respective groups are given above the* columns*

The overall patient-reported assessment of efficacy was rather positive. TTH patients rated efficacy slightly higher than migraine patients (97 vs. 93 % “good” or “very good”, respectively; p = 0.048; Fig. 2a, b).

Patients taking the APC combination for the first time rated efficacy slightly less positive, compared to patients who already had experience with the medication (90 vs. 97 % “good” or “very good”; p < 0.001; Fig. 2c, d).

### Tolerability and adverse events

**Fig. 3:**
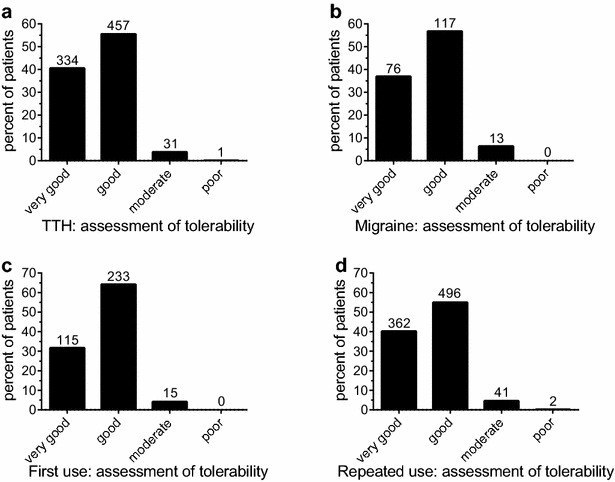
Patients’ assessment of tolerability. Assessment of tolerability for patients using the APC combination for the treatment of TTH (**a**) or migraine (**b**), as well as for patients taking the medication for the first time (**c**), compared to experienced users (**d**). See text for details. Numbers of patients for the respective groups are given above the* columns*

Overall tolerability was rated by 95 % of the patients as “good” or “very good”. No relevant differences were reported for TTH versus migraine (96 vs. 94 % “good” or “very good”; p = 0.34, Fig. 3a, b), as well as for first time compared to experienced users (96 vs. 95 % “good” or “very good”; p = 0.022; Fig. 3c, d).

No serious adverse drug reactions were reported during this survey. 95 % of the 143 adverse event reports stated intake of Thomapyrin^®^ INTENSIV for treating other than headache or migraine pain. Since in Germany the product is placed on the market solely for symptomatic treatment of headache or migraine (different from Thomapyrin^®^ CLASSIC), these reports were addressed as off-label use. Poor efficacy was reported by the remaining 5 %. In summary, no new safety information was retrieved from the survey.

## Discussion

Pharmacy-based patient surveys are valuable tools to gain information on patient demographic and characteristics of the treated complaints, as well as on the effects of investigated treatments as assessed by the patient. Especially in the context of OTC treatment, such surveys can provide insights which are otherwise hard to obtain and complement those of controlled clinical trials (Nieber and Lehmacher 2009).

### Patient characteristics/study population

This study investigated patients treating acute pain with two OTC available APC combination analgesics. The overall prevalence of headache is high (Pfaffenrath et al. 2009), and, therefore, it was not surprising that most of the patients reported headache or migraine as treated ailment (83 % in total). In line with published data, women are more frequently affected by headache and migraine, compared to men. Not unexpectedly, patients suffering from migraine reported more severe pain and more days with impairment of daily activities than headache patients.

### Pain anamnesis

In this survey, patients were asked to categorize their pain (as “headache”, “migraine”, or “other pain”). In addition, patients were asked to describe their headache characteristics according to ICHD-II in order to discriminate between TTH and migraine, and their personal assessment was compared to diagnoses according to ICHD-II (see Table 2). This analysis showed some discrepancies. Notable, only 68 % of patients with migraine reported unilateral pain. On the other hand, 26 % of the patients with TTH reported their pain to be throbbing. Other characteristics, however, appeared to be more specific. Nausea, photophobia or phonophobia were only reported by 10 % of those suffering from TTH, but by 96 % of the patients with migraine. Based on the data obtained in this study, it cannot be concluded whether patients treating their headaches with OTC analgesics are not sufficiently educated to judge migraine from TTH, or whether the extensive symptom overlap might have caused the difficulties in differentiating both types of headache. This is in line with data from a study which analysed diagnosis at first attendance in Italian headache centres, where only 26.8 % of the migraine patients had the previous correct diagnosis of migraine (Cevoli et al. 2009). Patient education may be helpful for recognizing migraine attacks early by the patients themselves because early treatment (“treat when mild”) might improve treatment response (D’Amico et al. 2006; Gendolla 2008). Asking the patient about treatment response and modify acute treatment if necessary may prevent progression into chronic migraine over time (Lipton et al. 2015).

### Treatment with the APC analgesic

The most prevalent reason for buying the APC product was recommendation by the pharmacist or friends/relatives (Table 3). In 2.8 % of the cases, the analgesic was recommended by a physician. In the guideline of the German, Austrian and Switzerland’s headache societies on the OTC treatment of episodic TTH and migraine, the APC combination was rated as “treatment of first choice”, and was the only analgesic obtaining a “highlighted recommendation on the basis of the analysed comparative studies” (Haag et al. 2011). Thus, there appears to be a gap between the evidence-based treatment guideline and recommendation practice by pharmacists and physicians.

It can be hypothesized that the higher burden induced by migraine affected the medication use in this study: Compared to patients with TTH, those suffering from migraine reported higher baseline pain levels, took the first analgesic dose earlier, on average the first dose was higher, and the same held true for the total number of tablets taken per pain event. Onset of pain relief appeared to occur later (Fig. 1). On the other hand, the patient ratings of efficacy did not reveal significant difference: More than 93 % of migraine patients rated the efficacy to be “good” or “very good”, compared to 97 % of patients with TTH (Fig. 2). Ratings for tolerability were similar for both patient groups (Fig. 3).

The results of open studies (like this survey) could be biased by patients’ expectations, as well as previous experience with the investigated medicinal products. In this survey, about 29 % of patients had no previous experience with the test products. A lower proportion of these patients reported pain relief within the first 15 min, but no relevant differences were observed after 30 min (Fig. 1). Assessment of efficacy was slightly in favour of patients with previous experience, but still 90 % of the APC-naive patients reported the efficacy to be “good” or “very good”, and assessment of tolerability was comparable (Fig. 2).

### Comparison with other investigations

Since pain is a highly individual experience, the patient assessment of efficacy (and tolerability) is a valuable measure of the individual treatment success of a given analgesic. Data from investigations on OTC doses of ibuprofen and acetylsalicylic acid for the treatment of migraine attacks with comparable methodology like this study have been reported. Here, 75.6 % of patients using ibuprofen to treat their migraine, and 76.8 % of patients using acetylsalicylic acid assessed the efficacy to be “good” or better (Goebel 2007; Krall 2007) compared to more than 90 % as reported in this study.

In two controlled clinical trials on treatment of acute migraine, 38.2 and 58.6 % patients taking ibuprofen reported “good” efficacy (Sandrini et al. 1998; Diener et al. 2004a, b); for acetylsalicylic acid these figures were between 32.5 and 45.6 % (Lange et al. 2000; Diener et al. 2004a, b). In the two trials on the APC combination 63 % of patients with migraine (Goldstein et al. 2005) and 73 % of patients with migraine or TTH (Diener et al. 2005) reported efficacy to be “good” or better. In the latter study patients suffering from TTH as well as migraine were included, revealing only very small differences in efficacy between these types of headache (72.0 vs. 76.6 %, respectively, data on file).

Thus, although methodologies of patient surveys and of controlled studies do differ, it appears that the (caffeine-containing) APC combination offers clinical benefit to a higher number of patients. This conclusion is also supported by meta-analyses on caffeine as co-analgesic (Derry et al. 2014, 2015).

## Conclusions

The main reason for purchasing APC products in the pharmacy are TTH or migraine. About a third of patients fulfilling the IHCD-II criteria for migraine failed to recognize their headache as migraine. This could be due to patients’ misconceptions about their pain, or the extensive symptom overlap between migraine and TTH. Education of patients about their headache disorder might be a way to improve treatment of migraine and TTH in future. Patients’ assessments of efficacy and tolerability of the investigated APC combinations showed that the investigated APC combinations are beneficial for the treatment of TTH and migraine headache. These data complement those of randomized clinical studies on such preparations.

Taken together this study shows that pharmacy-based surveys can provide valuable data on patients and their ailments. Moreover, they can broaden our knowledge on treatment of these with OTC medicinal products, and provide information on the individual patient experiences with the investigated medication.
